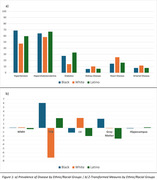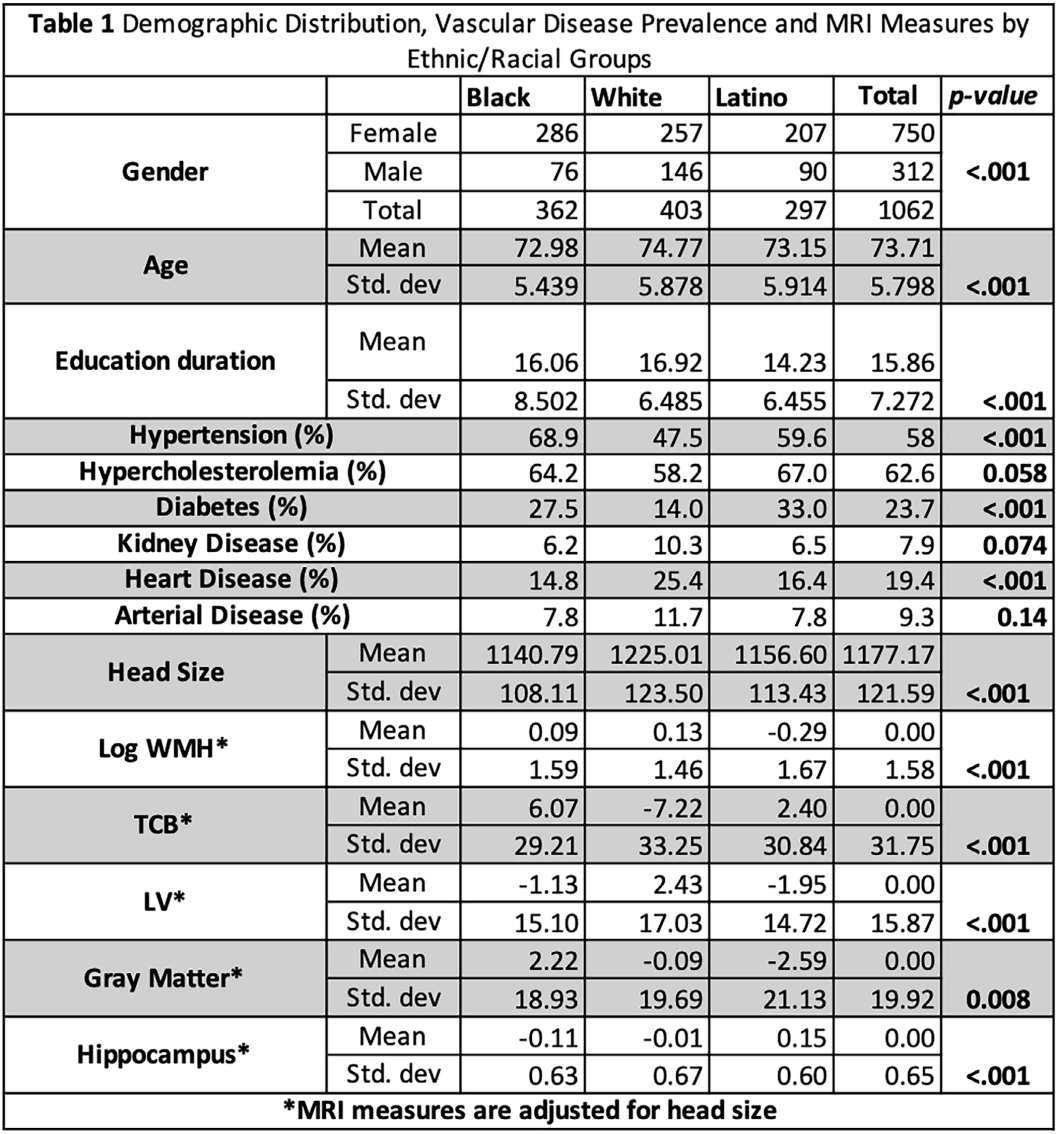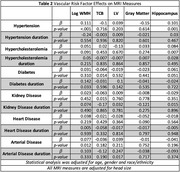# Vascular Risk Factors and Brain Structure in Diverse Vascular Cognitive Impairment and Dementia (DVCID) Study: Preliminary Findings

**DOI:** 10.1002/alz70856_103824

**Published:** 2025-12-25

**Authors:** Perla Mansour, Pauline Maillard, Kumar B Rajan, Lee‐Way Jin, Jason Hinman, David K Johnson, Danielle J. Harvey, Myriam Fornage, Charles S. DeCarli

**Affiliations:** ^1^ University of California, Davis, Davis, CA, USA; ^2^ Rush University Medical Center, Chicago, IL, USA; ^3^ University of California Los Angeles, Los Angeles, CA, USA; ^4^ The Brown Foundation Institute of Molecular Medicine, McGovern Medical School, The University of Texas Health Science Center at Houston, Houston, TX, USA; ^5^ UC Davis, Davis, CA, USA

## Abstract

**Background:**

The DVCID study, a prospective observational multicenter and multiethnic project, aims to understand the association between vascular disease and risk factors with the development of cognitive impairment and dementia.

**Method:**

For this preliminary analysis, the cohort consisted of 1,062 individuals with baseline MRI and vascular risk assessment. The cohort had a mean age of 73.7 ± 6 (range: 61‐91) years, and included 71% females, 34% Black, 38% White, and 28% Latino individuals, many of whom reported cognitive complaints (71%) or had mild cognitive impairment. Descriptive statistics and linear regression analyses were conducted on baseline MRI variables and vascular risk factors across racial and ethnic groups. WMH was log transformed and all MRI variables were adjusted for head size

**Result:**

We observed significant ethnic and racial differences in the prevalence of vascular risk factors, clinical diagnoses, and MRI measures (Table 1). In this diverse cohort, the prevalence of diabetes was high at 24%. Black participants exhibited the highest prevalence of hypertension at 68.9% and diabetes at 27.5%. White participants had the largest head size (1225 cc, *p* <0.001) and WMH volume at 0.13 (*p* <0.001), while Latino participants had the lowest WMH volume at ‐0.29 (*p* <0.001). Black participants had the highest mean brain tissue volume (TCB), whereas White participants had the lowest volume (6.07 vs ‐7.22, *p* <0.001). Latino participants showed the highest hippocampal volume, while Black participants had the lowest (0.15 vs ‐0.11, *p* <0.001). Hypertension was significantly associated with increased WMH (β= 0.111, *p* <0.001; Table 2) and increased hippocampal volume (β= 0.101, *p* = 0.001). Conversely, diabetes was associated with decreased TCB (β= ‐0.064, *p* = 0.001). The duration of diabetes (β=0.142, *p* = 0.033) and the presence of arterial disease (β=0.077, *p* = 0.012) were associated with larger WMH volumes. Longer durations of arterial disease were associated with larger lateral ventricle (LV) volumes (β= 0.247, *p* = 0.017).

**Conclusion:**

Preliminary analysis of baseline DVCID data found significant differences by race and ethnicity in vascular risk factors, disease prevalence, and MRI measures. Our study also found significant associations between hypertension and WMH. Further analysis is necessary to investigate the relationship between these factors and cognitive decline in the DVCID multiethnic study.